# Evaluating interventions to reduce behaviour associated with HCV reinfection in men who have sex with men: study protocol for a non-blinded, phase 2, randomised trial

**DOI:** 10.1186/s13063-023-07161-y

**Published:** 2023-03-15

**Authors:** Kris Hage, Anders Boyd, Udi Davidovich, Paul Zantkuijl, Elske Hoornenborg, Amy Matser, Ellen Generaal, Janke Schinkel, Eve Todesco, Marc van der Valk, Hayette Rougier, Karine Lacombe, Maria Prins, on behalf of the ICECREAM study group, S. Rebers, F. Pijnappel, H. van Eden, J. Stalenhoef, F. van Malsem, R. van Heerde, H. Nobel, W. Alers, L. Elsenburg, D. Verhagen, F. Lauw, M. van Wijk, J. den Hollander, A. Brouwer, E. Leyten, S. Wildenbeest, T. Mudrikova, M. van der Kerkhof, C. Kips, L. Flobbe, F. Mouthaan, S. Elzinga, D. Loomans, E. Ersan, K. Yap, K. de Jong, I. Peters, S. de Graaf, Ejay de Wit

**Affiliations:** 1 Department of Infectious Diseases, Public Health Service of Amsterdam, Amsterdam, the Netherlands; 2 Amsterdam UMC location University of Amsterdam, Infectious Diseases, Meibergdreef 9, Amsterdam, the Netherlands; 3 Amsterdam Institute for Infection and Immunity, Infectious Diseases, Amsterdam, the Netherlands; 4 Stichting hiv monitoring, Amsterdam, the Netherlands; 5 University of Amsterdam, Department of Psychology, Amsterdam, the Netherlands; 6 Soa Aids Nederland, Amsterdam, the Netherlands; 7 National Institute for Public Health and the Environment (RIVM), Centre for Infectious Disease Control (Cib), Bilthoven, the Netherlands; 8 Amsterdam UMC location University of Amsterdam, Medical Microbiology and Infection Prevention, Meibergdreef 9, Amsterdam, the Netherlands; 9 Sorbonne Université, INSERM, Institut Pierre Louis d’Epidémiologie et de Santé Publique (IPLESP), APHP, Hôpital Pitié-Salpêtrière, Laboratoire de virologie, Paris, France; 10 IMEA, Institut de Médecine et d’Épidémiologie Appliquée, Paris, France; 11 Faculté de médecine, Sorbonne Université, Inserm IPLESP, Hôpital St Antoine, APHP, Paris, France

**Keywords:** HCV, Reinfection, Randomised trial, Intervention, Risk behaviour

## Abstract

**Background:**

As highly effective therapy against hepatitis C virus (HCV) infection is available with rapid uptake, there is newfound optimism for HCV elimination. Nevertheless, certain key populations have a high risk of HCV reinfection, in particular men who have sex with men (MSM) in Western European countries. Modelling data indicate that HCV elimination will not be feasible without reduction in risk behaviour, thus supporting the need for effective interventions aimed at reducing risk behaviour and preventing reinfections in MSM.

**Methods:**

The ICECREAM study is an international, multi-centred, phase 2, 3-arm randomised trial comparing run-in and intervention periods enrolling MSM with a history of a cured or spontaneously cleared HCV infection. Individuals are followed in routine care for 6 months (i.e. run-in period) and then randomly allocated (1:1:1) to one of the following: a tailored, interactive online risk-reduction behavioural intervention, a validated home-based HCV-RNA self-sampling test service using dried blood spots, or a combination of both. After randomisation, individuals are followed every 6 months until 18 months (i.e. intervention period). Interventions are delivered in addition to standard of care. Online questionnaire measuring risk behaviour over the past 6 months is administered at every visit. The primary outcome is the proportion at risk of HCV infection during run-in versus intervention periods assessed by using the HCV-MOSAIC risk score. The risk score consists of six self-reported HCV-related risk behaviours. Secondary outcomes include incidence of HCV reinfection, changes in the individual risk behaviour items and changes in sexual well-being since changes in sexual behaviour may have an impact on sexual experience. Two hundred forty-six MSM aged 18 years or older will be invited to participate.

**Discussion:**

The ICECREAM study is a trial aimed at establishing interventions that could effectively decrease the incidence of HCV re-infection in MSM with a previous HCV infection. By offering an online behavioural risk-reduction intervention and HCV-RNA self-sampling, both of which are aimed to influence risk behaviour, we are able to provide products to at-risk MSM that could further reduce population-level HCV incidence and ultimately help reach HCV micro-elimination.

**Trial registration:**

ClinicalTrials.gov NCT04156945. Registered on November 8, 2019

## Administrative information

**Table:** 

Title	Interventions to Curb hEpatitis C REinfections Among Men who have sex with men (the ICECREAM study)
Trial registration	Dutch IRB registration number: NL68718.018.19 French IRB registration number: 2022-A00533-40 www.ClinicalTrials.gov identifier: NCT04156945
Protocol version	Version 7.0, 17/03/2022
Funding	The Netherlands Organisation for Health Research and Development (ZonMw; grant number 522004006), the ANRS | Maladies infectieuses émergentes (grant number ECTZ108101) and the Research and Development Foundation of the Public Health Service of Amsterdam (grant number 50-52200-98-558).
Author details	1 Department of Infectious Diseases, Public Health Service of Amsterdam, Amsterdam, the Netherlands 2 Amsterdam UMC location University of Amsterdam, Infectious Diseases, Meibergdreef 9, Amsterdam the Netherlands 3 Amsterdam Institute for Infection and Immunity, Infectious Diseases, Amsterdam, the Netherlands 4 Stichting hiv monitoring, Amsterdam, the Netherlands 5 University of Amsterdam, Department of Psychology, Amsterdam, the Netherlands 6 Soa Aids Nederland, Amsterdam, the Netherlands 7 National Institute for Public Health and the Environment (RIVM), Centre for Infectious Disease Control (Cib), Bilthoven, the Netherlands 8 Amsterdam UMC location University of Amsterdam, Medical Microbiology and Infection Prevention, Meibergdreef 9, Amsterdam, the Netherlands 9 Sorbonne Université, INSERM, Institut Pierre Louis d’Epidémiologie et de Santé Publique (IPLESP), APHP, Hôpital Pitié-Salpêtrière, Laboratoire de virologie, Paris, France 10 IMEA, Institut de Médecine et d’Épidémiologie Appliquée, Paris, France 11 Faculté de médecine, Sorbonne Université, Inserm IPLESP, Hôpital St Antoine, APHP, Paris, France
Name and contact information for the trial sponsor	The Netherlands: Public Health Service of Amsterdam Nieuwe Achtergracht 100, 1018 WT Amsterdam Telephone : 020 555 5911 France: Institut de Médecine et d'Épidémiologie Appliquée et Tropicale Hôpital Bichat, 46 rue Henri Huchard, 75018 Paris Telephone: 01 40 25 69 58 Email: imea.dpo@imea.fr
Role of sponsor	The funders are independent from the trial. These funding entities have no role in the design of the trial and do not have any role during the execution, management, analyses of the project, or in the interpretation of results. The ICECREAM writing committee will write the report and will decide where to submit the report for publication, independent of the trial sponsors and funders.

## Introduction

### Background and rationale {6a}

Worldwide, an estimated 58 million people are chronically infected with the hepatitis C virus (HCV) with an incidence of 1.5 infections per year. HCV is a bloodborne virus and is mainly transmitted through exposure to unsafe injecting health care practices (e.g. use of contaminated needles and syringes), unsafe injecting drug use and sexual contact [1, 2]. Since 2000, outbreaks of sexually transmitted infections (STI) with HCV have been reported among men who have sex with men (MSM) with human immunodeficiency virus (HIV) [3, 4]. When left untreated, HCV can cause liver damage representing a major cause of morbidity and mortality [5, 6]. The availability of highly effective oral therapy against HCV, namely direct-acting antivirals (DAA), with cure rates exceeding 95% has led the World Health Organization (WHO) to set goals for HCV elimination by 2030: an 80% reduction in new chronic HCV infections and 65% reduction in HCV-related mortality from 2015, a target that is far from being reached [7].

In Western European countries, such as France and the Netherlands, new HCV infections are typically found in MSM with HIV. Before DAA became widely available, incidence of primary HCV infection stabilised around 2009 and did not markedly decline among MSM with HIV [4, 8–10]. Among MSM without HIV, emerging data show that the prevalence and incidence of HCV infection have been on the rise, possibly due to increased uptake of pre-exposure prophylaxis (PrEP) and increasing overlap in sexual networks [11–13]. Since unrestricted DAA became available, overall HCV incidence has sharply decreased [10, 14, 15]. Of great concern is that the risk of HCV reinfection is still high [12, 13, 16]. Increasingly overlapping sexual networks between MSM with and without HIV might put MSM without HIV also at risk of HCV (re) infection and spread to the larger population of MSM without HIV cannot be excluded [17, 18].

Given the genetic diversity of the virus, the progress of developing a successful HCV vaccine has been slow [19]. Modelling data indicate that in a setting with high DAA uptake among MSM, HCV elimination without the availability of an effective vaccine would not be feasible without a reduction in risk behaviour [20, 21]. In addition, this reduction could be more difficult to achieve as the new highly effective and well-tolerated treatments might result in a decreased perceived threat of HCV, especially in those at risk of HCV reinfection [22, 23]. This and the costs related to treatment highlight the urgent need for effective interventions aimed at reducing risk behaviour and preventing reinfections among MSM. To the best of our knowledge, behavioural interventions aimed at reducing risk behaviour for HCV infection have only been studied within the Swiss HCVree trial and have shown effectiveness and feasibility [24, 25]. As an example, more frequent testing for HCV might potentially influence HCV-related risk behaviour [20]. Home-based self-sampling and testing, for instance, may offer advantages compared to conventional HCV testing, as it has shown to increase convenience, perceived control over the testing procedure, patient autonomy and control over the individual’s own health [26, 27]. By increasing HCV risk awareness, additional HCV testing could reduce HCV-related risk behaviour. On the other hand, additional testing for which the test result is negative could also enhance risk-taking behaviour, as the individuals receiving these results may assume that their risk of HCV transmission is low. Unfortunately, most rapid HCV tests available are limited because they are unable to differentiate previous HCV exposure (i.e. HCV antibodies) from active infection (i.e. HCV antibodies and RNA) [28]. A recent project in Amsterdam, the Netherlands, using home-based HCV-RNA self-sampling dried blood spots (DBS) has shown that this strategy is successful in diagnosing HCV infections among MSM [27].

This project will evaluate two interventions (i.e. an online behavioural intervention and a home-based HCV testing intervention), alone or in combination among MSM with and without HIV who have a history of a cured or spontaneously cleared HCV infection. It is intended that these interventions could possibly contribute to achieve HCV elimination.

### Objectives {7}

The primary objective is to investigate whether a behavioural and testing intervention, alone or in combination, has an effect on self-reported behaviours associated with HCV acquisition in MSM with a previous HCV infection.

The secondary objectives are to evaluate the effect of an online behavioural and testing intervention, alone or in combination, on HCV reinfection incidence, STI incidence, and sexual well-being.

### Trial design {8}

**Fig. 1:**
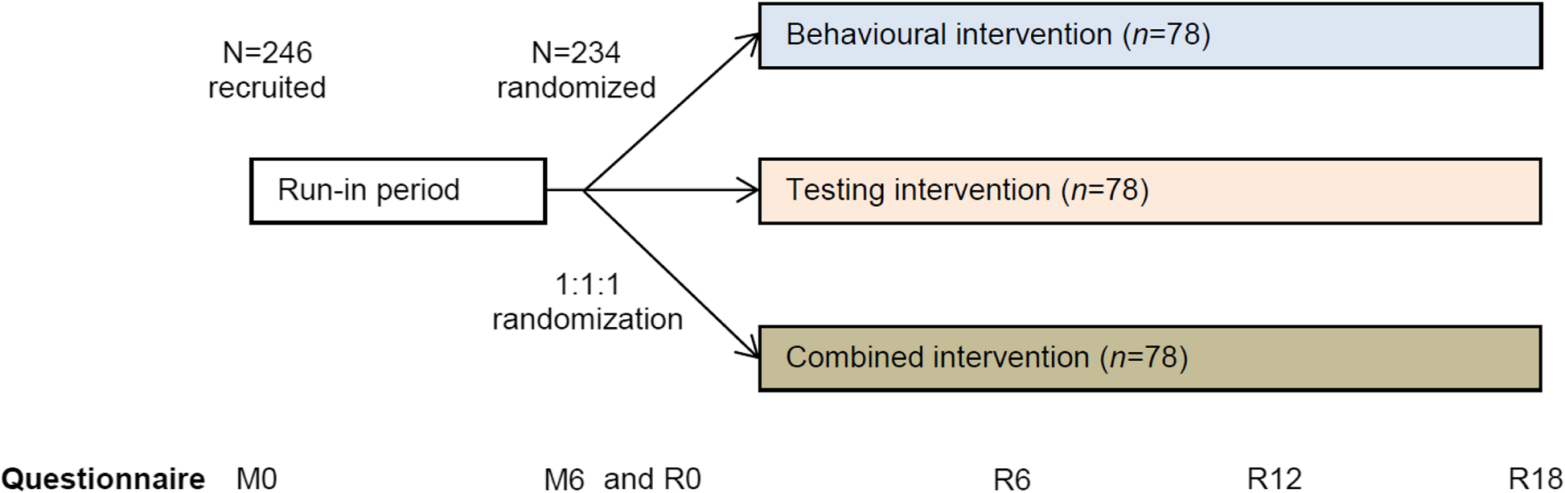
Illustration of the study design of the ICECREAM study. *Abbreviations:* M0, month 0; M6, month 6; R0, randomisation months 0; R6, randomisation month 6; R12, randomisation month 12; R18, randomisation month 18

This study is an international, multi-centred phase 2, 3-arm, randomised trial (RT) comparing run-in and intervention periods to evaluate the effect of an online behavioural intervention, a home-based sampling for testing intervention, or both on self-reported behaviours associated with HCV acquisition (Fig. 1). The first 6 months of the trial includes follow-up with no intervention (termed the “run-in” period) during which participants receive standard care (see the “Relevant concomitant care permitted or prohibited during the trial {11d}” section). At 6 months, participants are randomly assigned, with an allocation ratio of 1:1:1, to one of the following three arms: arm I consists of a targeted, online behavioural intervention developed as part of the project; arm II consists of an additional participant-initiated, home-based HCV-RNA self-sampling test service using DBS; and arm III consists of a combination of intervention I and II. Participants continue the intervention for 18 months (termed the “intervention” period) in addition to standard care. During both the run-in and intervention periods, five online questionnaires measuring risk behaviour over the past 6 months are administered (i.e. at months 0, 6, 12, 18 and 24 of the study).

## Methods

### Study setting {9}

Recruitment takes place at fourteen sites: HIV treatment centres, centres for sexual health (CSH; for participants in the Netherlands), and free-of-charge centres for information, diagnosis and testing (CeGIDD; for participants in France) located in Amsterdam, the Hague, Rotterdam and Utrecht, the Netherlands and Paris, France. These centres include: Onze Lieve Vrouwe Gasthuis; Amsterdam UMC, location AMC; DC Clinic Lairesse; Medical Centre Jan van Goyen; Amsterdam UMC, location VUmc (all located in Amsterdam, the Netherlands); Maasstad Ziekenhuis (Rotterdam, the Netherlands); Haaglanden Medical Centre (the Hague, the Netherlands); University Medical Centre Utrecht (Utrecht, the Netherlands); Service de maladies infectieuses et tropicales, Hôpital Saint-Antoine; Service de maladies infectieuses et tropicales, Hôpital La Pitié-Salpêtrière; Service de maladies infectieuses et tropicales, Hôpital Tenon; Le Centre 190; Maison Chemin Vert (all located in Paris, France).

### Eligibility criteria {10}

Participants are included in this study if they meet the following criteria: ≥18 years of age; previously cured or spontaneously cleared HCV infection (i.e. positive HCV-RNA test and/or positive anti-HCV antibody in the past with currently negative HCV-RNA); self-reported MSM; attending care at an HIV treatment centre (for participants with HIV) or a CSH/CeGIDD (for participants without HIV); sufficient understanding of Dutch or English (for participants in the Netherlands) or French (for participants in France); accept to be contacted by telephone; have health coverage within the national healthcare system (for participants in France); and have access to the internet and an e-mail messaging service.

Participants are excluded if they have any one of the following: acute or chronic HCV infection, receiving HCV treatment, suspected non-compliance with study procedures, being under legal guardianship (for participants in France), not able or incapable to provide informed consent, and participation in another study offering an HCV testing and/or an intervention targeting behaviours associated with risk of acquiring HCV. Individuals who are investigators or otherwise dependent persons are also not included in the study.

### Who will take informed consent? {26a}

Treating physicians and nurses at the study centres propose the study to individuals meeting inclusion criteria. Information is given both verbally and in a written information brochure. There is an adequate opportunity provided to each individual for asking questions. Only until verbal and written informed consent is obtained will the individual be included in the study.

### Additional consent provisions for collection and use of participant data and biological specimens {26b}

All participants are asked to give consent to link HCV test data results from routine care and to test stored blood samples. These stored samples are collected during regular clinical visits at the HIV treatment centre or CSH/CeGIDD during participation in the ICECREAM study and are stored at the laboratory in accordance with legal regulations. Retrospective testing of stored samples takes place at the end of the study only if the participant has not been tested for HCV during routine care in the 6 months prior to study completion.

## Interventions

### Explanation for the choice of comparators {6b}

Initially, we considered a randomised trial in which a control arm with no intervention was compared to three interventions. We consulted with members of the MSM community who expressed concerns around the unattractiveness of a control arm and the potential for increased rates of not only non-participation in the study, but also loss to follow-up in a control arm (personal communication, Paul Zantkuijl, Soa Aids Nederland). We decided that the risk of a non-representative or small sample and differential loss to follow-up between arms would outweigh any benefit from using a control arm. Hence, we opted instead to compare a 6-month run-in period without an intervention to a follow-up period of 18 months with an intervention (Fig. 1). The primary endpoint is the proportion at risk of HCV infection which are compared between the run-in and intervention periods, within each arm.

### Intervention description {11a}

The study includes three arms involving two interventions, either alone or combined. Follow-up during the intervention period ends after 18 months. The interventions are as follows:

#### Arm I: behavioural intervention

This arm consists of a completely web-based behavioural intervention. This online tailored intervention is based on the principles of the Information-Motivation-Behavioural (IMB) skills model for behavioural change [29]. The model explains health behaviour through the possession of sufficient information, motivation and behavioural skills to execute health behaviour. Based on these principles, the behavioural intervention addresses knowledge gaps and barriers of motivation and skills for applying HCV-related risk reduction strategies. The intervention consists of several tailored modules to counteract the barriers for reducing sexual risk behaviour and is partially based on the e-health-assisted counselling intervention used in the Swiss HCVree trial [25].

**Fig. 2:**
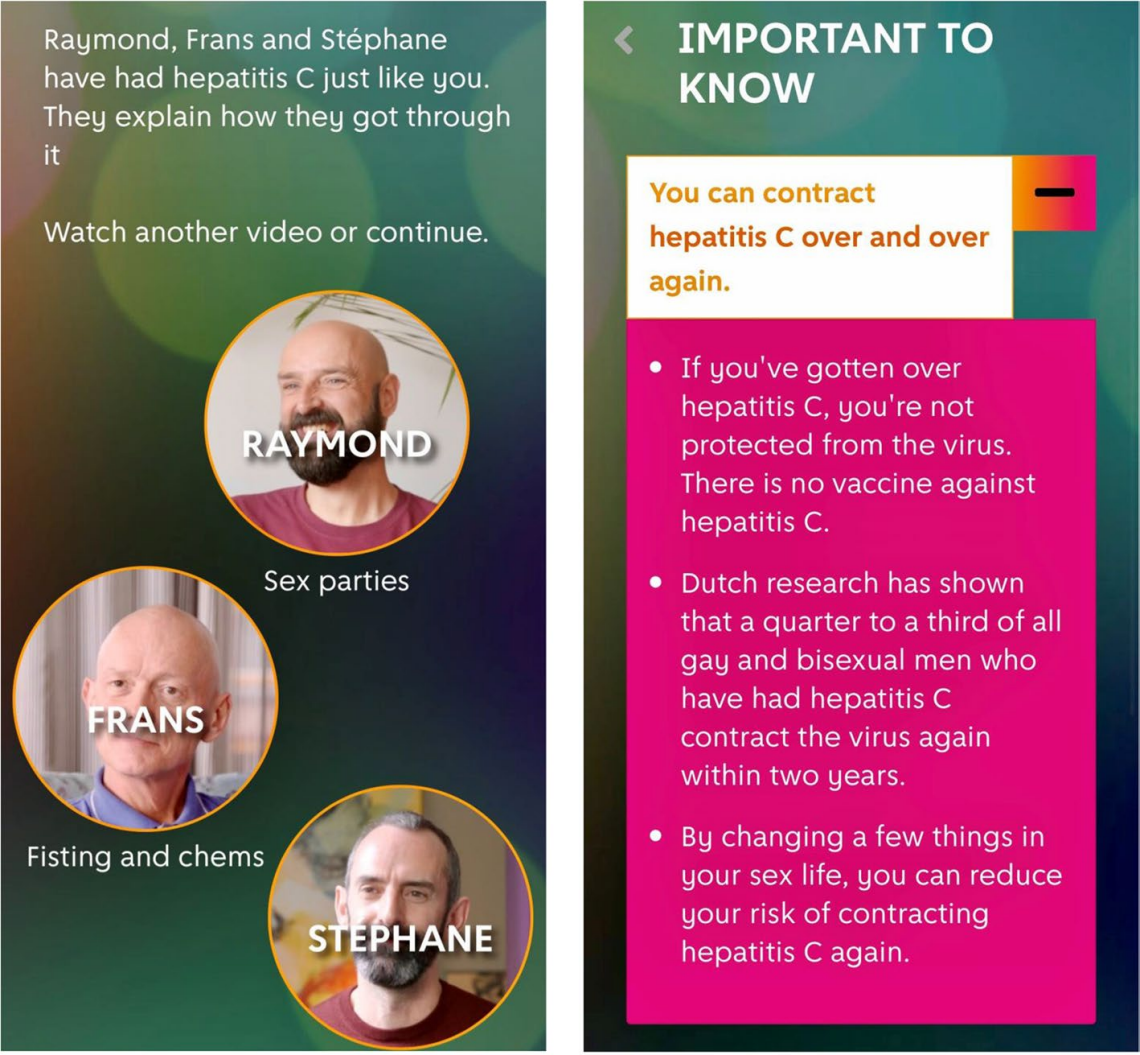
Screenshots of the online tool of the ICECREAM study (left from Module 1 “*Hepatitis C & I*” and right from Module 2 “*What is important to know*”)

The content of the intervention consists of interactive questions, tailored text-based modules, and videos addressing information, motivation, and behavioural skills (Fig. 2). It comprises four modules:

Module 1, “Hepatitis C & I”, focuses on self-reflection and exploring the intrinsic motivation of participants to reduce the risk of HCV infection using filmed role models. The videos are based on modelling principles of behaviour where peers tell real stories about their experiences and challenges with HCV-related risk behaviours and how they addressed these challenges. After watching the video(s), participants answer self-reflective questions and questions regarding personal motivation to reduce their HCV risk.

Module 2, “What is important to know?”, focuses on increasing HCV-related knowledge. Participants receive tailored information about modes of transmission and a summary of personal HCV risk factors based on the participant’s earlier disclosed risk behaviour in the study questionnaire (i.e. the items of the HCV-MOSAIC risk score and other HCV-related risk factors).

Module 3, “Making my plan”, identifies the necessary steps to achieve behavioural goals, lending to a personalised risk reduction plan that is created by the participant online. The participant will be able to choose personal goals tailored by their answers to the study questionnaire. They are also offered an option to formulate their own goal(s). Relevant solutions to overcome personal psychosocial/cognitive barriers are then offered according to the chosen goal(s). For instance, there is a module to promote skills efficacy that provides tools for basic communication between sex partners, such as suggested conversation openers and discussion scenarios.

Module 4, “Evaluating my plan”, focuses on evaluating the risk reduction plan. The participant will be able to reflect on their risk reduction plan, whether it achieved their desired goals and their level of satisfaction with it. Subsequently, participants can modify their goal(s) if desired and are encouraged to formulate a new action plan and return for evaluation after three months again in case their goal(s) has/have not been achieved.

Modules 1–3 are offered immediately after randomisation and can be done together without any time constraints. Three months after completing the third module, participants receive an email notification asking them to evaluate their goal(s) and, when appropriate, are asked to create a new plan (i.e. Module 4). When a new plan is formulated, the participant is asked to evaluate it three months after and so on. Participants also have the opportunity to return to modules 1–3 at any moment.

#### Arm II: Home-based self-sampling testing intervention

This arm consists of a participant-initiated, home-based HCV-RNA self-sampling test service that is delivered in addition to routine care. The service offers free-of-charge HCV-RNA tests which are delivered at the address provided by the participant (in the Netherlands and France) or can be picked up at the Public Health Service of Amsterdam (in the Netherlands) and can be used during the remaining 18 months of the trial at any moment. Participants are advised to use the free-of-charge HCV-RNA tests in between visits at their HIV treatment centre or CSH/CeGIDD, particularly when they perceive themselves at risk for HCV reinfection. Self-sampling is performed on DBS. The sampling kit contains paper instructions on how to collect blood droplets, two contact-activated lancets (2.0-mm BD Microtainer), two band aids, a DBS card (Whatman Protein Saver 903 card), alcohol wipe, gauze wipe, grip seal bag, desiccant sachet and a medical return envelope (UN 3373). In addition, paper instructions are included along with an online demonstration video to assist blood collection, which is available on the study website (www.icecreamstudy.nl) [26]. The DBS card is then sent to a centralised laboratory for HCV-RNA testing. Participants are informed of their test result by secured email and, if HCV-RNA positive, are immediately offered linkage to clinical care. DBS self-sampling has been found to be a feasible and valid technique for the detection of HCV-RNA [26].

#### Arm III: behavioural and home-based testing intervention combined

This arm combines the behavioural and testing intervention.

### Criteria for discontinuing or modifying allocated interventions {11b}

There are no plans to discontinue or modify allocated interventions during the trial.

### Strategies to improve adherence to interventions {11c}

When an individual does not complete the study questionnaire, they receive two email notifications (10 days apart) to complete it. For individuals randomised to arms including the online behavioural intervention, the intervention is offered in a tailored fashion, meaning that it is continuously adapted by the answers given in the study questionnaire and personal needs of each user. Since participants receive information related to their situation, the intervention becomes more concise and relevant and thus helps increase adherence to the intervention [30]. Participants who do not complete all modules of the intervention, as intended, receive an email reminder to visit the website.

For individuals randomised to arms including the at-home sampling intervention, reminders to use the tests are included in the emails that invite the participant to complete the study questionnaires at study months 12 and 18 (i.e. months 6 and 12 of the intervention period).

### Relevant concomitant care permitted or prohibited during the trial {11d}

The interventions are delivered in addition to standard care. In the Netherlands, MSM with HIV who report HCV-related risk-taking behaviour are supposed to be screened for HCV-RNA at least annually at the HIV clinic and screening is mostly based on elevated alanine transaminase levels (ALT) in the blood [31]. MSM with HIV visiting the SHC in Amsterdam are also screened for HCV at least once a year. In regions outside Amsterdam in the Netherlands, MSM with HIV are screened for elevated ALT every 6 months and those with elevated ALT levels and a history of HCV infection are tested for HCV-RNA [32]. MSM using PrEP are screened every 6 to 12 months, depending on reported risk [33]. MSM without HIV who are not using PrEP are not routinely tested for HCV, unless they receive a notification that a sex partner tested positive for HCV, refuse to test for HIV or in the presence of a Lymphogranuloma Venereum infection [32]. In France, it is recommended to regularly screen individuals at risk for HCV, including MSM with and without HIV [34].

### Provisions for post-trial care {30}

No post-trial care for study participants will be provided.

### Outcomes {12}

#### Primary study outcome(s)

The primary outcome is the proportion at risk of HCV infection (as determined by the HCV-MOSAIC score) during the run-in versus intervention periods. The score is calculated by summing up the *β*-coefficients specific to six self-reported risk factors when present in the previous 6 months: (i) receptive condomless anal sex (*β*=1.1), (ii) sharing sex toys (*β*=1.2), (iii) unprotected fisting (*β*=0.9), (iv) injecting drug use (*β*=1.4), (v) sharing snorting equipment during nasally-administered drug use (*β*=1.0), and (vi) ulcerative STI (*β*=1.4). An HCV-MOSAIC risk score of ≥2.0 defines an individual at risk of HCV infection, as validated for acute (primary) HCV infection and reinfection in HIV-positive MSM [35, 36]. This information is obtained using the answers from the study questionnaire every 6 months.

#### Secondary study outcome(s)

The secondary outcomes are as follows:

1. Incidence rate of HCV reinfections, both self-reported and laboratory-confirmed, defined as the number of cases divided by the total person-years of follow-up at risk for reinfection.
2. Incidence rate of any STI, only self-reported, defined as the number of chlamydia, gonorrhoea, genital herpes and/or syphilis infections divided by the total person-years of follow-up.
3. Changes in HCV-related risk behaviour during run-in versus intervention period, more specifically:
  - Changes in the number of sex partners.
  - Changes in the number of condomless anal sex acts with casual partners.
  - Changes in the individual items of the HCV-MOSAIC risk score.
4. Changes in the proportion of individuals disinfecting sex toys, skin and possible contaminated surfaces (e.g. plastic sheets, sling, bench).
5. Changes in sexual well-being score.

#### Other study outcomes

Other study parameters include the adherence to intervention-related endpoints: number of HCV tests used, from home-based sampling provided by the study or otherwise (e.g. at the HIV clinic or general practitioner); and behavioural intervention-related endpoints: frequency of use, time spent on the intervention and proportion of individuals completing all modules of the intervention, type of goals set in the behavioural intervention and usability and acceptability of the behavioural intervention.

### Participant timeline {13}

Participants are followed for 24 months and are asked to fill in questionnaires every 6 months from study enrollment. Enrollment takes place at the month 0 study visit and randomisation at the month 6 study visit. The intervention continues during the month 12, 18, and 24 study visits, at which only questionnaires are administered. The schedule of study procedures is described in Table 1.

**Table 1:** Schedule of enrollment and study procedures in the ICECREAM study

Study procedures	Inclusion	Month 0 or M0	Month 6 or M6 and R0	Month 12 or R6	Month 18 or R12	Month 24 or R18	After the end of the study
Eligibility check	X						
Informed consent	X						
Questionnaire		X	X	X	X	X	
Online randomisation			X				
Behavioural intervention			X	X	X		
Testing intervention			X	X	X		
Combined intervention			X	X	X		
HCV RNA testing in a stored blood sample^a^							X

^a^If (1) the participant did not receive an HCV RNA test in the 6 months prior to the end of the study period, (2) there is a blood sample available for HCV RNA testing stored at the laboratory of the participating site, and (3) the participant provided consent for retrospective HCV RNA testing

*Abbreviations*: *HCV* hepatitis C virus, *M0* month 0, *M6* month 6, *R0* randomisation month 0, *R6* randomisation month 6, *R12* randomisation month 12, *R18* randomisation month 18, *RNA* ribonucleic acid

### Sample size {14}

We aim to have a minimum of 78 individuals per arm (total: 234) who complete at least one study questionnaire during the intervention period. Assuming a maximum drop-out rate of 5% during the run-in period, 246 participants in total are needed to be enrolled (Fig. 1). We simulated power under varying proportions at risk of HCV infection during the run-in period and absolute risk reduction during the intervention. With a sample size of 78 in each arm, we would have 80% power to demonstrate a statistically significant difference, at a type 1 error of 0.05, of a >22% reduction in the primary end-point from a 60% proportion at risk of HCV infection during the run-in period.

### Recruitment {15}

Treating physicians and nurses at the participating study centres enrol participants. Participants are also recruited through targeted ads on gay dating apps (i.e. Scruff, Grindr and Recon).

## Assignment of interventions: allocation

### Sequence generation {16a}

Lists of randomly permuted blocks assigning six interventions (i.e. two from each of the three arms) are generated from a computer. The rationale for blocking is to reduce the predictability of a random sequence. Randomisation is not stratified on any factor.

### Concealment mechanism {16b}

Randomisation takes place via an online, centralised system. Research staff do not have access to the randomisation sequence nor do they know the arms randomly assigned to the individuals before a given participant is recruited.

### Implementation {16c}

The allocation sequences are generated from a computer. The program to generate these sequences was authored by a data manager at the Public Health Service of Amsterdam (Amsterdam, the Netherlands). During the study, individuals who reach the 6-month visit receive an email stating to which arm they have been randomised.

## Assignment of interventions: blinding

### Who will be blinded {17a}

Participants are unblinded to the study arms, since the interventions involve distinct procedures that cannot be blinded. The treating physicians and nurses are not formally informed of the assigned intervention; however, participants are allowed to discuss their interventions with their care providers and hence treating physicians and nurses are not considered to be blinded. During analysis, the data analyst is to conduct analysis while being blinded to the intervention arm.

### Procedures for unblinding if needed {17b}

The analyst is unblinded to the study interventions as soon as all analysis in the most recent Statistical Analysis Plan (SAP) is completed. A copy of the SAP will be uploaded to the trials register once finalised.

## Data collection and management

### Plans for assessment and collection of outcomes {18a}

Patient demographic data is collected at baseline. In total, participants are asked to fill in five online questionnaires, each occurring within an interval of 6 months, containing personal questions about sexual behaviour, drug use and clinical data (for more details see Table 2). The questionnaires were developed using LimeSurvey software (LimeSurvey GmbH, Hamburg, Germany). Data on the use of the behavioural intervention (i.e. web-based application), including which of the components were used and how often they were used, are collected from the study website using login data obtained from a backend log. Data on the number of free HCV home-based self-sampling test(s) used and their test results are collected from either the Castor EDC platform (in the Netherlands) or from a centralised laboratory database (in France). Additional data on markers and treatment of HCV reinfection (i.e. HCV-RNA test date(s), test results and name of HCV treatment regimens along with their dates of initiation and discontinuation) are collected from laboratory and clinical files of participating sites and for participants from centres in the Netherlands who provided consent for data linkage, from the Dutch HIV monitoring foundation (*Stichting HIV Monitoring*). Additional HCV-RNA blood testing will retrospectively be performed to test for HCV reinfection if (1) the participant did not receive an HCV-RNA test in the 6 months prior to the end of the study period, (2) there is a blood sample available for HCV-RNA testing stored at the laboratory of the participating site, and (3) the participant provided consent for retrospective HCV-RNA testing.

**Table 2:** Collection of outcomes in the ICECREAM study trial

	Questionnaire 1 (month 0 or M0)	Questionnaire 2 (month 6 or M6 and R0)	Questionnaire 3 (month 12 or R6)	Questionnaire 4 (month 18 or R12)	Questionnaire 5 (month 24 or R18)
Demographic characteristics	X				
HIV-status and PrEP-use	X	X	X	X	X
Previous HCV infections and treatment	X	X	X	X	X
Sexual relationships and sexual behaviour^a^	X	X	X	X	X
STI testing and testing outcomes^b^	X	X	X	X	X
Alcohol and drug use	X	X	X	X	X
Sexual well-being	X	X	X	X	X
Motivation to reduce risk behaviour	X	X	X	X	X
HCV threat appraisal	X	X	X	X	X
Influence COVID-19 measures on sexual behaviour	X	X	X	X	X
Influence monkeypox on sexual behaviour	X	X	X		
Testing intervention-related endpoints^c^			X		
Behavioural intervention-related endpoints^d^			X	X	X
Usability and acceptability of testing and behavioural intervention			X	X	X

*Abbreviations*: *HCV* hepatitis C virus, *HIV* human immunodeficiency virus, *M0* month 0, *M6* month 6, *PrEP* pre-exposure prophylaxis, *R0* randomisation month 0, *R6* randomisation month 6, *R12* randomisation month 12, *R18* randomisation month 18, *RNA* ribonucleic acid, *STI* sexually transmitted infection

^a^Including variables included in the HCV-MOSAIC risk score, number of sex partners and condomless anal sex acts with casual partners, and changes in disinfecting behaviour (self-reported)

^b^Including testing for chlamydia, gonorrhoea, genital herpes and/or syphilis (self-reported)

^c^Number of free HCV tests used and number of HCV-positive test results

^d^Including website statistics (e.g. frequency of use, time spent on the intervention and proportion completing all modules of the intervention, types of goals set and proportion individuals reporting change in risk behaviour identified in the goal setting module

### Plans to promote participant retention and complete follow-up {18b}

As only one site visit is required at the start of the study (i.e. to sign informed consent) and all further study procedures take place online or at home, this is a low-threshold study for participation. The relatively limited amount of time needed to participate (e.g. 20-min baseline questionnaire, 60 min for the entire behavioural intervention) is intended to maintain study retention and help participants complete follow-up.

### Data management {19}

Data management from both French and Dutch centres is centralised at the Public Health Service of Amsterdam (Amsterdam, the Netherlands). The data from the questionnaires, the behavioural intervention and HCV test results (from both the at-home sampling intervention and routine care) are imported into a Research SQL database. From this SQL database, data can be exported into other formats for analysis.

### Confidentiality {27}

To protect the privacy of the participants, all data and body materials are coded. Participant data are stored in two separate databases: one containing the code and directly identifiable personal data (i.e. participant identification code list) and the other containing the code and study data (e.g. sexual risk behaviour data). The participant identification code list is safeguarded by a key and is only accessible to the study coordinator and data manager, if necessary. When participants are assigned to additional testing or combined intervention, the data relating to the home-based tests are also stored separately from the personally identifiable information.

### Plans for collection, laboratory evaluation and storage of biological specimens for genetic or molecular analysis in this trial/future use {33}

DBS samples from the home-based self-sampling intervention are kept in the laboratory of the Amsterdam UMC, location AMC (for samples collected in the Netherlands) or La Pitié-Salpêtrière Hospital (for samples collected in France) until 1 year after the end of the project. These samples may later be used for identifying HCV transmission clusters through phylogenetic analysis. No other biological specimens are stored for this study.

## Statistical methods

### Statistical methods for primary and secondary outcomes {20a}

The six items of the HCV-MOSAIC risk score are obtained using the answers from the study questionnaire every 6 months. The proportion achieving the primary outcome is summarised at months 6, 12, 18 and 24 study questionnaires within each study arm. The probability of the primary outcome is compared between the run-in (month 6) and intervention periods (month 12, month 18, month 24) using a mixed-effect logistic regression model. In this model, each individual serves as their own control and between-individual differences at baseline are accounted for using a random intercept. Run-in versus intervention period odds ratios (ORs) and their 95% confidence intervals (CI) are estimated and stratified on the study arm, allowing us to identify interventions with significant differences. No multivariable adjustments are applied. Additional analyses are planned to be conducted in which ORs between arms are compared by including and testing an interaction term between period and arm in the model (two arms at a time, for a possible three comparisons). No *p*-value adjustments are made for multiple comparisons to avoid unnecessary correction on the possibly underpowered test for interaction [37].

Incidence rates of HCV reinfections and STIs are calculated at the end of follow-up. Incidence of HCV reinfection is examined during the run-in and intervention periods of the RT. Considering that few HCV reinfections are likely to occur, we intend to analyse differences in periods, along with associated risk factors, using Bayesian exponential survival regression models with non to weakly informative *a priori* distributions [38, 39].

For all other secondary study parameters, continuous variables are summarised using means or medians and categorical variables using counts and percentages at each study visit. Changes over time are described for HCV-related risk behaviour, disinfection behaviour and sexual well-being. We use statistical regression methods specific to the endpoint (logistic for binary outcomes, linear for continuous variables), corrected for repeated measurements within individuals (using mixed-effect methods) to investigate changes between run-in and intervention periods and associated determinants. Outcomes are also compared across the three arms. Descriptive statistical analyses are performed to describe study population characteristics, intervention-related outcomes (e.g. use of services, acceptability and usability) and the number of tests, home-based or otherwise, performed.

### Interim analyses {21b}

No interim analysis is planned.

### Methods for additional analyses (e.g. subgroup analyses) {20b}

Subgroup analyses are conducted to determine whether individuals with certain demographic or clinical characteristics are more likely to have a decrease in HCV-MOSAIC risk score.

### Methods in analysis to handle protocol non-adherence and any statistical methods to handle missing data {20c}

Analyses are performed by intention to treat (ITT) and per protocol (PP). Both analyses are to include individuals completing the run-in phase (month 6). ITT analysis is defined by including all observations, while assuming that any individual who was lost to follow-up did not achieve the primary endpoint. PP analysis is defined by including all available observations, while excluding observations after an individual has been lost to follow-up.

### Plans to give access to the full protocol, participant-level data and statistical code {31c}

The protocol is currently restricted to study investigators and staff. It is to be made publicly available as a supplementary appendix of the article in which these results are published. Upon completion of the study, participant-level data for own research purposes can be requested by submitting a research proposal to the Principal Investigator (Maria Prins). Statistical code can be provided upon request to the trial statistician (Anders Boyd).

## Oversight and monitoring

### Composition of the coordinating centre and trial steering committee {5b}

The Scientific Committee is composed of clinicians, virologists, epidemiologists, and methodologists involved in the present protocol. As regulations and administrative aspects vary between the Netherlands and France, each country has their own Scientific Committee. Information is regularly exchanged between Scientific Committees. The goal of the Scientific Committee is to oversee that research is properly conducted, on a scientific, ethical, and logistical level. It regularly ensures that the research is conducted as planned and that the protocol is respected, notably with regards to subject safety. A scientific advisory board composed of a community member, virologist, behavioural scientist and a statistician is also involved in the present protocol, from which advice was given on the feasibility and continuation of the study during the COVID-19 pandemic.

### Composition of the data monitoring committee, its role and reporting structure {21a}

This trial includes minimally invasive interventions that are unlikely to incur adverse events. The trial is also designed to assess the sustainability of any effect on behaviour associated with the intervention. These conditions would make it unnecessary for a data monitoring committee to convene and intervene.

### Adverse event reporting and harms {22}

Only one site visit is required at the start of the study and all further study procedures take place online or at home. As such, serious adverse events or serious adverse reactions cannot be readily monitored. No harmful effects of the interventions are remotely expected during participation.

### Plans for communicating important protocol amendments to relevant parties (e.g. trial participants, ethical committees) {25}

All amendments are to be notified to the Medical Research Ethics Committee (METC; Dutch IRB registration number NL68718.018.19) in the Netherlands and the Committee for the Protection of Persons (CPP; French IRB registration number 2022-A00533-40) in France. All substantial amendments are to be notified to the METC or CPP and to the competent authority. Non-substantial amendments are not notified to the METC or CPP and the competent authority, but are recorded and filed by the study sponsor. If amendments affect participants in any way, they are informed about the changes and if needed, additional consent is to be requested and registered.

### Dissemination plans {31a}

Study outcomes are to be presented at scientific conferences and published in the most appropriate, scientific, peer-reviewed journals. Authorship is determined by the guidelines from the International Committee of Medical Journal Editors [40]. In addition, participants, members of the community and relevant stakeholders will be informed in layman’s terms about the study results through newsletters. If significant decreases in risk behaviour occur between pre- and post-intervention periods for a given intervention, were are planning to make the intervention available outside the research setting.

## Discussion

The incidence of HCV reinfection among MSM shortly after HCV clearance remains high, suggesting that some MSM may not have sufficient understanding, skills or motivation to implement HCV risk reduction strategies or are not motivated to do so. To the best of our knowledge, this is the first trial with an evaluation of randomly assigned interventions aimed at influencing risk behaviour for HCV reinfection in MSM. Our suggested approach is diverse, combining cognitive reappraisal and skills enhancement to influence risk behaviour as well as disrupting onward HCV transmission through RNA testing. Because of the high risk for HCV reinfection and the required reduction in risk behaviour to reach WHO elimination goals, this RT provides a unique possibility to evaluate innovative interventions or strategies to reduce HCV risk behaviour and ultimately reach HCV elimination.

While previous studies on sexual risk reduction for HCV have mainly focused on promoting the use of condoms, there have been few evaluations of behavioural interventions focusing on more extensive behaviours associated with HCV infection among MSM. The Swiss HCVree trial offered a theory-based intervention consisting of four e-health-assisted counselling sessions, carried out by trained nurses and supported by an e-health tool, to individuals who reported condomless sex with casual partners [41]. The design of this intervention was leveraged from a previous intervention to prevent HIV infection [42]. The structure of the behavioural intervention to be evaluated in the ICECREAM study is partially based on this e-health-assisted counselling intervention. For the current project, we opted for a completely web-based intervention without the use of counsellors, nurses or other healthcare professionals, as such an intervention may be more straightforward to implement in routine care. In addition, our goal is to also focus on risk reduction strategies other than condom use and tailor them according to the cognitive and behavioural risk profile of each participant. In this sense, we mimic the counselling effect that would have been observed from the HCVree trial.

The potential for facilitated HCV testing, such as home-based self-sampling, as a means to influence behavioural risk, was previously often ignored. The NoMoreC project is one of the first studies evaluating a home-based HCV-RNA self-sampling test service, offered to all individuals at risk of HCV reinfection [27]. The results from this study indicated high usability, acceptability and satisfaction among its users. A user-initiated testing service increases convenience, perceived control over the testing procedure and patient autonomy and control over their own health [26, 27]. We assume that uptake of the tests will be high when participants perceive themselves at risk for HCV reinfection. The influence of additional testing on HCV-related risk behaviour remains uncertain: additional testing may increase awareness about HCV risk and promote the implementation of risk reduction strategies among users. However, when additional testing shows no presence of HCV-RNA particles in the blood, risk-taking behaviour could become enhanced as users may assume that their risk of HCV transmission is low. Nevertheless, additional home-based HCV-RNA self-sampling could have the added advantage of early detection and treatment for HCV, thereby limiting onward transmission of the virus.

Other study designs for the presented trial were entertained. A parallel design with a control arm including no intervention was not appealing enough for potential participants, as deemed by representatives of the community. A study comparing only interventions would be challenging, as the differences between arms are expected to be minimal. To achieve sufficient power for this type of study, it would require an unrealistic number of study participants given the small sample of MSM at risk of HCV reinfection in the Netherlands and France who are willing to adapt risk behaviours associated with HCV. The use of a stepped-wedge study design would also be an alternative, yet would require too many individuals and some centres beginning the intervention at later timepoints would not have been able to provide enough follow-up to adequately measure the sustainability of the intervention. Hence, the use of a design with a run-in versus intervention comparison offered the most valid means of evaluating the effectiveness of the intervention, while taking the suggestions from the community into account.

The run-in versus intervention design does, however, pose a few limitations. First, individuals are required to arrive at the month-6 study timepoint before being randomised. This selection bias could render a group of more motivated participants to continue the intervention, although the resulting population is likely to represent the target population of users who would benefit more from these services. Second, temporality could play a role in changes in behaviour associated with HCV. It is assumed that these behaviours do not normally display seasonality, but the continuing pandemic of the severe acute respiratory virus-covariant 2 (SARS-CoV-2) and mpox outbreak may influence certain sexual behaviours [43]. We have included a few items in the questionnaires about the influence of SARS-CoV-2 lockdown measures and mpox on sexual behaviour and plan to evaluate this issue more closely during the analysis phase.

One difficulty with estimating the required sample size to demonstrate a significant difference is that no initial data on the effects of our interventions, with respect to the behaviours included in the HCV-MOSAIC score, were available. Modelling studies have consistently shown in similar epidemiological contexts as the Netherlands and metropolitan France that at least a 20% reduction in behaviours associated with HCV is needed to reach WHO goals of HCV elimination in MSM [44]. We then decided to base our sample size calculation on this more epidemiologically meaningful change in behaviours from the run-in versus intervention periods. Given the complexity of the model, we simulated varying study conditions and arrived at a sample size of 234, or 78 participants per arm, when also considering the rate of loss to follow-up.

The primary comparison in endpoints is between the run-in and intervention periods within arms and not between arms. It could be argued that randomisation is unnecessary and that participants could choose their own intervention, which may increase intervention uptake and effectivity. We did, however, decide against this and to randomly assign individuals to a study arm. Randomisation ensures that participants of all arms are likely to belong to the same target population of MSM (i.e. exchangeability) and that the interventions can be applied to this target population. In addition, this may give a better reflection of intervention uptake when interventions are being implemented in real-world settings.

In conclusion, we have presented the protocol of a trial aimed at establishing interventions, from a public health perspective, that are needed to sufficiently reduce behaviours associated with the risk of HCV. If significant decreases in risk behaviour occur between run-in versus intervention periods for a given intervention, this intervention could contribute to reducing incident reinfections and achieving HCV micro-elimination. Any intervention able to demonstrate this level of effectiveness would be likely implemented into standard of care. Regardless, the interventions developed herein may help in offering information on prevention or facilitating access to HCV-RNA testing.

## Trial status

The study initially started in November 2019. However, it was forced to discontinue recruitment in March 2020 and restart in September 2021 due to the COVID-19 pandemic. Recruitment of participants is expected to be completed by mid-2023. The anticipated study completion date is June 2025.

## Data Availability

Data may be available upon reasonable request to the Principal Investigator (Maria Prins) and approval from the Scientific Committee.

## Abbreviations

ANRS: The National Agency of Research on AIDS and viral hepatitis
CI: Confidence Interval
CPP: Committee for the Protection of Persons
DAA: Direct-Acting Antivirals
DBS: Dried blood spots
HCV: Hepatitis C virus
HIV: Human immunodeficiency virus
IMB model: Information-Motivation-Behavioural skills model
ICECREAM: Interventions to Curb hEpatitis C Reinfections Among Men who have sex with men
IRB: Institutional Review Boards
ITT: Intention to treat
METC: Medical research ethics committee (MREC); in Dutch: medisch ethische toetsing commissie (METC)
MOSAIC: MSM Observational Study of Acute Infection with hepatitis C
MSM: Men who have sex with men
OR: Odds ratio
PP: Per protocol
PrEP: Pre-exposure prophylaxis
RNA: Ribonucleic acid
RT: Randomised trial
SAP: Statistical Analysis Plan
SARS-CoV-2: Severe acute respiratory virus-covariant 2
STI: Sexually transmitted infection
WHO: World Health Organization
ZonMw: The Netherlands Organisation for Health Research and Development (in Dutch: Nederlandse organisatie voor gezondheidsonderzoek en zorginnovatie)

## References

[1] Prati D (2006). Transmission of hepatitis C virus by blood transfusions and other medical procedures: a global review. J Hepatol.

[2] van de Laar TJ, Matthews GV, Prins M, Danta M (2010). Acute hepatitis C in HIV-infected men who have sex with men: an emerging sexually transmitted infection. AIDS.

[3] Hagan H, Jordan AE, Neurer J, Cleland CM (2015). Incidence of sexually transmitted hepatitis C virus infection in HIV-positive men who have sex with men. AIDS.

[4] van Santen DK, van der Helm JJ, Del Amo J, Meyer L, D'Arminio Monforte A, Price M (2017). Lack of decline in hepatitis C virus incidence among HIV-positive men who have sex with men during 1990-2014. J Hepatol.

[5] Alavi M, Grebely J, Hajarizadeh B, Amin J, Larney S, Law MG (2018). Mortality trends among people with hepatitis B and C: a population-based linkage study, 1993-2012. BMC Infect Dis.

[6] Dicker D, Nguyen G, Abate D, Hassen Abate K, Abay S, Abbafati C (2018). Global, regional, and national age-sex-specific mortality and life expectancy, 1950-2017: a systematic analysis for the Global Burden of Disease Study 2017. Lancet.

[7] World Health Organization (WHO) (2017). Global hepatitis report.

[8] Vanhommerig JW, Stolte IG, Lambers FA, Geskus RB, van de Laar TJ, Bruisten SM (2014). Stabilizing incidence of hepatitis C virus infection among men who have sex with men in Amsterdam. J Acquir Immune Defic Syndr.

[9] Hullegie SJ, van den Berk GEL, Leyten EMS, Arends JE, Lauw FN, van der Meer JTM (2016). Acute hepatitis C in the Netherlands: characteristics of the epidemic in 2014. Clin Microbiol Infect.

[10] Smit C, Boyd A, Rijnders BJA, van de Laar TJW, Leyten EM, Bierman WF (2021). HCV micro-elimination in individuals with HIV in the Netherlands 4 years after universal access to direct-acting antivirals: a retrospective cohort study. Lancet HIV.

[11] Hoornenborg E, Achterbergh RCA, Schim van der Loeff MF, Davidovich U, Hogewoning A, de Vries HJC (2017). MSM starting preexposure prophylaxis are at risk of hepatitis C virus infection. AIDS.

[12] Hoornenborg E, Coyer L, Boyd A, Achterbergh RCA, Schim van der Loeff MF, Bruisten S (2020). High incidence of HCV in HIV-negative men who have sex with men using pre-exposure prophylaxis. J Hepatol.

[13] Ramiere C, Charre C, Miailhes P, Bailly F, Radenne S, Uhres AC (2019). Patterns of Hepatitis C Virus Transmission in Human Immunodeficiency Virus (HIV)-infected and HIV-negative Men Who Have Sex With Men. Clin Infect Dis.

[14] Boerekamps A, van den Berk GE, Lauw FN, Leyten EM, van Kasteren ME, van Eeden A (2018). Declining Hepatitis C Virus (HCV) Incidence in Dutch Human Immunodeficiency Virus-Positive Men Who Have Sex With Men After Unrestricted Access to HCV Therapy. Clin Infect Dis.

[15] Garvey LJ, Cooke GS, Smith C, Stingone C, Ghosh I, Dakshina S (2021). Decline in Hepatitis C Virus (HCV) Incidence in Men Who Have Sex With Men Living With Human Immunodeficiency Virus: Progress to HCV Microelimination in the United Kingdom?. Clin Infect Dis.

[16] Hosseini-Hooshyar S, Hajarizadeh B, Bajis S, Law M, Janjua NZ, Fierer DS (2022). Risk of hepatitis C reinfection following successful therapy among people living with HIV: a global systematic review, meta-analysis, and meta-regression. Lancet HIV.

[17] Vanhommerig JW, Bezemer D, Molenkamp R, Van Sighem AI, Smit C, Arends JE (2017). Limited overlap between phylogenetic HIV and hepatitis C virus clusters illustrates the dynamic sexual network structure of Dutch HIV-infected MSM. AIDS.

[18] MacGregor L, Martin NK, Mukandavire C, Hickson F, Weatherburn P, Hickman M (2017). Behavioural, not biological, factors drive the HCV epidemic among HIV-positive MSM: HCV and HIV modelling analysis including HCV treatment-as-prevention impact. Int J Epidemiol.

[19] Hepatology TLG (2021). The hunt for a vaccine for hepatitis C virus continues. Lancet Gastroenterol Hepatol.

[20] Martin NK, Thornton A, Hickman M, Sabin C, Nelson M, Cooke GS (2016). Can Hepatitis C Virus (HCV) Direct-Acting Antiviral Treatment as Prevention Reverse the HCV Epidemic Among Men Who Have Sex With Men in the United Kingdom? Epidemiological and Modeling Insights. Clin Infect Dis.

[21] Salazar-Vizcaya L, Kouyos RD, Zahnd C, Wandeler G, Battegay M, Darling KE (2016). Hepatitis C virus transmission among human immunodeficiency virus-infected men who have sex with men: Modeling the effect of behavioral and treatment interventions. Hepatology.

[22] Lambers F, van der Veldt W, Prins M, Davidovich U, study M. (2018). Changing the odds: motives for and barriers to reducing HCV-related sexual risk behaviour among HIV-infected MSM previously infected with HCV. BMC Infect Dis.

[23] Berenguer J, Gil-Martin A, Jarrin I, Montes ML, Dominguez L, Aldamiz-Echevarria T (2019). Reinfection by hepatitis C virus following effective all-oral direct-acting antiviral drug therapy in HIV/hepatitis C virus coinfected individuals. AIDS.

[24] Braun DL, Hampel B, Ledergerber B, Grube C, Nguyen H, Kunzler-Heule P (2021). A Treatment-as-Prevention Trial to Eliminate Hepatitis C Among Men Who Have Sex With Men Living With Human Immunodeficiency Virus (HIV) in the Swiss HIV Cohort Study. Clin Infect Dis.

[25] Kunzler-Heule P, Fierz K, Schmidt AJ, Rasi M, Bogdanovic J, Kocher A (2021). Response to a sexual risk reduction intervention provided in combination with hepatitis C treatment by HIV/HCV co-infected men who have sex with men: a reflexive thematic analysis. BMC Infect Dis.

[26] Prinsenberg T, Rebers S, Boyd A, Zuure F, Prins M, van der Valk M (2020). Dried blood spot self-sampling at home is a feasible technique for hepatitis C RNA detection. PLoS One.

[27] Prinsenberg T, Schinkel J, Zantkuijl P, Davidovich U, Prins M, van der Valk M (2022). Internet-guided HCV-RNA testing: A promising tool to achieve hepatitis C micro-elimination among men who have sex with men. J Viral Hepat.

[28] Grebely J, Applegate TL, Cunningham P, Feld JJ (2017). Hepatitis C point-of-care diagnostics: in search of a single visit diagnosis. Expert Rev Mol Diagn.

[29] Fisher JD, Fisher WA, Williams SS, Malloy TE (1994). Empirical tests of an information-motivation-behavioral skills model of AIDS-preventive behavior with gay men and heterosexual university students. Health Psychol.

[30] Krebs P, Prochaska JO, Rossi JS (2010). A meta-analysis of computer-tailored interventions for health behavior change. Prev Med.

[31] European AIDS Clinical Society (EACS). Guidelines; 2021. Available from: https://www.eacsociety.org/guidelines/eacs-guidelines/. Updated Version 11.0. Accessed 19 Sep 2022.

[32] Rijksintituut voor Volksgezondheid en Milieu (RIVM). Draaiboek voor Centra Seksuele Gezondheid in de Publieke Gezondheidszorg. Bilthoven: Rijksinstituut voor Volksgezondheid en Milieu (RIVM); 2018.

[33] Nederlandse Vereniging van HIV Behandelaren (NVHB). Nederlandse multidisciplinaire richtlijn Pre-expositie profylaxe (PrEP) ter preventie van hiv update; 2022. Available from: https://www.soaaids.nl/nl/professionals/themas/prep. Accessed 14 Jul 2022.

[34] Mallatt A, Bureau C, Fontaine H, Hanslik B, Hézode C, de Lédinghen V (2018). Recommandations AFEF pour l'élimination de l'infection par le virus de l'hépatite C en France: Association Francaise pour l'étude de foie (AFEF).

[35] Newsum AM, Stolte IG, van der Meer JT, Schinkel J, van der Valk M, Vanhommerig JW (2017). Development and validation of the HCV-MOSAIC risk score to assist testing for acute hepatitis C virus (HCV) infection in HIV-infected men who have sex with men (MSM). Euro Surveill.

[36] Hage K, van de Kerkhof M, Boyd A, Newsum A, Matser A, van der Valk M, et al. Screening for hepatitis C virus reinfection using a behaviour-based risk score among HIV-positive men who have sex with men - Abstract poster 69 Brussels, Belgium: EASL Elimination Meeting; 2022. Available from: https://easl.eu/event/viral-hepatitis-elimination-2022/abstract-information/.

[37] Rothman KJ (2010). Curbing type I and type II errors. Eur J Epidemiol.

[38] Young J, Rossi C, Gill J, Walmsley S, Cooper C, Cox J (2017). Risk Factors for Hepatitis C Virus Reinfection After Sustained Virologic Response in Patients Coinfected With HIV. Clin Infect Dis.

[39] Newsum AM, Matser A, Schinkel J, van der Valk M, Brinkman K, van Eeden A (2021). Incidence of HCV reinfection among HIV-positive MSM and its association with sexual risk behavior: a longitudinal analysis. Clin Infect Dis.

[40] International Committee of Medical Journal Editors (ICMJE). Recommendations for the Conduct, Reporting, Editing, and Publication of Scholarly Work in Medical Journals Updated May 2022. Accessed Jul 14 2022. Available from: https://www.icmje.org/recommendations/. 10.12771/emj.2024.e48 PMC12093628 40704003

[41] Braun DL, Hampel B, Kouyos R, Nguyen H, Shah C, Flepp M (2019). High Cure Rates With Grazoprevir-Elbasvir With or Without Ribavirin Guided by Genotypic Resistance Testing Among Human Immunodeficiency Virus/Hepatitis C Virus-coinfected Men Who Have Sex With Men. Clin Infect Dis.

[42] Apers H, Vuylsteke B, Loos J, Smekens T, Deblonde J, Van Beckhoven D (2020). Development and evaluation of an HIV-testing intervention for primary care: protocol for a mixed methods study. JMIR Res Protoc.

[43] Jongen VW, Zimmermann HML, Boyd A, Hoornenborg E, van den Elshout MAM, Davidovich U (2021). Transient changes in preexposure prophylaxis use and daily sexual behavior after the implementation of COVID-19 restrictions among men who have sex with men. J Acquir Immune Defic Syndr.

[44] Martin NK, Jansen K, An der Heiden M, Boesecke C, Boyd A, Schewe K (2019). Eliminating Hepatitis C Virus among human immunodeficiency virus-infected men who have sex with men in berlin: a modeling analysis. J Infect Dis.

